# Subsequent Primary Hematologic Malignancies in a 21‐Year‐Old Retinoblastoma Survivor: Case Report Study

**DOI:** 10.1002/cnr2.70411

**Published:** 2025-11-27

**Authors:** Elham Karimi, Shalaleh Abbasnezhad, Maedeh Arabpour, Sepideh Abdollahi, Mohammad Biglari, Marjan Yaghmaie

**Affiliations:** ^1^ Department of Medical Genetics, School of Medicine Tehran University of Medical Sciences Tehran Iran; ^2^ Research Institute for Oncology, Hematology and Cell Therapy Tehran University of Medical Sciences Tehran Iran; ^3^ Hematology, Oncology and Stem Cell Transplantation Research Center, Research Institute for Oncology, Hematology and Cell Therapy Tehran University of Medical Sciences Tehran Iran

**Keywords:** chemotherapy, retinoblastoma, SMNs, TRb

## Abstract

**Background:**

Retinoblastoma (RB) is a malignant eye tumor that predominantly affects children. Although survival rates have improved significantly due to advancements in treatment, subsequent malignant neoplasms (SMNs) continue to be major causes of death in both heritable and non‐heritable RB cases. These SMNs are often associated with mutations in the RB1 gene, as well as the effects of radiotherapy or chemotherapy. There are no previous reports of a nonhereditary RB survivor developing three sequential hematologic malignancies (AML, lymphoma, and ALL) over 20 years. Most secondary primary cancers (SPCs) in RB survivors are solid tumors, such as osteosarcoma, soft tissue sarcoma, and melanoma, with hematologic malignancies being far less common, especially as third or subsequent primary tumors.

**Case:**

We report a case of a 21‐year‐old Iranian male who developed multiple distinct hematologic malignancies following retinoblastoma treatment. Using NGS, Sanger sequencing, and bioinformatic analysis, the possibility of germline mutations was surveyed.

**Conclusion:**

Germline changes associated with malignancies were examined using next‐generation sequencing (NGS). There were no germline alterations discovered, suggesting no predisposition to develop cancer. Three pathogenic/likely pathogenic heterozygous variants were found in the patient by carrier screening. Absence of germline RB1 mutations or other hereditary cancer syndromes implicates treatment‐related factors (chemotherapy/radiotherapy) as the primary driver of sequential malignancies. Nonhereditary retinoblastoma (RB) survivors have a lower risk of secondary malignancies (SMNs) compared to their hereditary counterparts. Chemotherapy, especially alkylating agents, increases the risk of secondary acute myelogenous leukemia (AML) and other leukemias and lymphomas due to its mutagenic effects and genetic factors. Although RB survivors rarely develop secondary cancers, the limited patient numbers and short follow‐up periods may influence SPC risk assessments. Continuous monitoring and personalized follow‐up care are crucial for managing long‐term risks in these survivors. This research emphasizes the essential importance of ongoing monitoring and follow‐up for survivors of retinoblastoma (RB) to identify and address secondary malignancies (SMNs), improve the management of long‐term complications, and enhance both life expectancy and quality of life.

## 1 Introduction

Retinoblastoma (RB) affects approximately 9000 children worldwide each year, an aggressive primary intraocular cancer of childhood [1, 2]. Biallelic loss‐of‐function variations in the RB1 gene on chromosome 13 cause both heritable and sporadic forms of RB [3]. Besides a germline mutation in one allele, patients with heritable RB acquire a de novo oncogenic variant in the other allele, while in the nonhereditary form, both RB1 allele mutations are acquired [3]. Patients with a hereditary form of RB develop bilateral and/or multifocal disease at an earlier age compared to the sporadic form [4]. Advanced therapeutic management has improved the survival of RB patients dramatically [5]; however, patients with hereditary RB have a predisposition for second primary cancers (SPCs), which is the major cause of mortality in these patients [6, 7, 8]. The most commonly observed SPCs are pineoblastoma, osteosarcoma, and soft tissue sarcoma, but due to the improved survival of RB patients, they are at increased risk of various types of cancers, including carcinoma, melanoma, and leukemia/lymphoma in adulthood [9, 10, 11]. Therapeutic modalities may play a crucial role in predisposition to the type and incidence of SPCs. Due to the significantly high occurrence of SPCs in patients treated with radiation, the primary treatment changed to chemotherapy in the late 1990s [12, 13]. Sarcomas are significantly associated with radiation, whereas there might be an increased risk for leukemia in chemotherapy‐treated individuals as well [14, 15, 16, 17]. Further studies are needed to understand the association of hematologic SPCs with germ‐line pathogenic variants and treatment strategies, particularly secondary acute myeloid leukemia or myelodysplastic syndrome [18]. Implementing robust surveillance strategies for the early detection and management of subsequent malignancies is essential [19]. For individuals who have had external beam radiation therapy (EBRT), this risk is especially high [20]. The cumulative incidence of future malignancies in patients receiving radiation therapy has fluctuated, reaching 8.4% at 18 years and 36% at 50 years after diagnosis [21]. The present study reports the long‐term risk of second malignancies in a 21‐year‐old Iranian male who survived retinoblastoma. This case report presents a unique and unprecedented instance of a nonhereditary RB survivor who developed three sequential hematologic malignancies—acute myeloid leukemia (AML), lymphoma, and acute lymphoblastic leukemia (ALL)—over 20 years following initial RB treatment. To our knowledge, no previous reports document such a pattern of multiple distinct hematologic secondary malignancies in a nonhereditary RB patient. By integrating next‐generation sequencing and comprehensive genetic analyses, which revealed no germline *RB1* or other hereditary cancer syndrome mutations, we confirmed the nonhereditary nature of the patient's retinoblastoma. This case is unusual and highlights the potential long‐term mutagenic effects of chemotherapy and radiotherapy even in the absence of germline predisposition. It also expands the known spectrum of secondary hematologic malignancies in nonhereditary RB survivors and underscores the need for individualized, lifelong surveillance strategies to inform future care guidelines.

## 2 Methods

### 2.1 Case Description

A 21‐year‐old Iranian male from Qom province presented to the Shariati Hospital in Tehran. He was diagnosed with multiple distinct malignancies: retinoblastoma at the first year, acute myeloid leukemia (AML) at age 7, and T lymphoblastic lymphoma and acute lymphoblastic leukemia (ALL) at the age of 20. He had no family history of cancer except for his maternal grandfather, who died from a brain tumor. He had received chemotherapy and radiation therapy (until he was 7 years old).

He was first diagnosed with retinoblastoma when he was 11 months old. His parents noticed eye malalignment along with swelling and redness in his right eye. Leucocoria was identified at examination, and a thorough ophthalmic exam revealed a large 3 cm mass in the right eye extending to the optic nerve. Biopsy was done, which showed malignant small round cells with optic nerve invasion. Immunohistochemistry revealed positive staining for GFAP, SOX‐2, and pRB, confirming the diagnosis of retinoblastoma. No genetic testing was done at that time. Neoadjuvant chemotherapy with carboplatin, etoposide, and vincristine combination was initiated and continued for 7 months. After maximal response (tumor size 2.5 cm), radiotherapy was performed using external beam radiation to further increase the chance of vision preservation. Unfortunately, the tumor was not responsive enough, which made vision preservation impossible. Thus, right eye enucleation was performed, and no further treatment was pursued. The patient was under surveillance, and later on, he presented with echymosis, epistaxis, and fatigue when he was 7 years old. A complete blood count revealed profound thrombocytopenia, anemia, and leukocytosis. Bone marrow aspiration and biopsy were performed, and an acute myelogenous leukemia (AML) diagnosis was verified. He received a so‐called “7 + 3” chemotherapy with continuous cytarabine infusion for 7 days and daunorubicin infusion for 3 days. Complete remission was achieved at day 21, and he further received high‐dose cytarabine as consolidation chemotherapy. The disease remained in remission, and no relapse was detected. Regular follow‐ups were conducted over 5 years. At the age of 21, he presented with fever, fatigue, and pancytopenia. He had cervical, axillary, and mediastinal lymphadenopathy. A biopsy of the axillary lymph node revealed T‐cell lymphoblastic lymphoma. After bone marrow aspiration and biopsy, marrow lymphomatous involvement was identified, and the diagnosis of acute T‐cell lymphoblastic leukemia/lymphoma was confirmed. Cerebrospinal fluid was not involved, and no other extramedullary disease was present. According to the simultaneous involvement of bone marrow and lymph nodes (T‐lymphoblastic leukemia/lymphoma), combination chemotherapy was chosen to benefit both components. He was treated with chemotherapy consisting of cyclophosphamide, vincristine, doxorubicin, and prednisolone (CHOP regimen) along with intrathecal methotrexate and cytarabine as central nervous system prophylaxis. Repeat bone marrow biopsy at the 28th day showed complete remission. Computed tomography (CT) scan at this time also showed resolution of lymphadenopathies. Treatment continued with mercaptopurine, vincristine, and methotrexate consolidation. Minimal residual disease (MRD) evaluation was performed regularly with established remission. According to the T‐ALL diagnosis, he was selected for hematopoietic stem cell transplantation (HSCT). Testing for Fanconi anemia (chromosomal breakage) was done with a negative result. The latest MRD was negative 3 weeks before allogeneic HSCT. He then received HSCT from his full‐match brother after the so‐called Flu‐Bu4 conditioning regimen. Graft‐versus‐host disease (GVHD) prophylaxis was cyclosporin and methotrexate. He is now 15 months post‐treatment, and no disease recurrence has been identified.

### 2.2 Sample Collection and Processing

The peripheral whole blood sample was collected in EDTA‐containing tubes following informed consent, according to the institutional guidelines and ethical approvals. The QIAamp DNA Mini Kit was used to extract genomic DNA from whole blood following the manufacturer's instructions. A NanoDrop spectrophotometer (Thermo Fisher Scientific, USA) was used to measure the extracted DNA's concentration and purity, and gel electrophoresis was used to further confirm the DNA integrity, guaranteeing that the sample satisfied the quality requirements for further sequencing. Illumina platforms were used for whole‐exome sequencing (Illumina Inc., CA, USA). Target enrichment and DNA library preparation were carried out using the Agilent SureSelect V6‐post Capture/Target Enrichment Kit following the manufacturer's guidelines. The manufacturer's instructions for DNA fragmentation, end‐repair, adapter ligation, and amplification were followed in the preparation of DNA libraries. Following that, the libraries were sequenced on Illumina platforms using paired‐end read lengths of 150 bp, resulting in an average coverage depth of 100× throughout the exome.

### 2.3 Bioinformatic Analysis

The conventional bioinformatics workflow was used to process the raw sequencing reads. In short, the Burrows‐Wheeler Aligner program was used to align the reads to the human reference genome (GRCh38 assembly). Utilizing the Genome Analysis Toolkit (GATK) program, variant calling was carried out. To find potentially harmful mutations, variants were annotated using the variant annotation program ANNOVAR. To concentrate on mutations associated with hematological malignancies, additional filtering was used.

### 2.4 Validation and Interpretation

To verify the existence of clinically significant mutations, Sanger sequencing on an ABI 3730 DNA Analyzer (Applied Biosystems) was used to validate key variants found using WES. The patient's diagnosis and the body of current literature and databases, such as ClinVar, COSMIC, and dbSNP, were taken into consideration when assessing the clinical significance of these mutations.

## 3 Result

This case report presents a 21‐year‐old male patient with a history of three distinct malignancies: retinoblastoma, acute myeloid leukemia (AML), and acute lymphoblastic leukemia/lymphoma, diagnosed at different ages. DNA was obtained from the peripheral blood of the patient before undergoing bone marrow transplantation. The donor was his full sibling, who was a perfect HLA match. Cells from the donor were used for allogeneic transplantation. Low‐resolution HLA typing revealed the following alleles for the patient: A02,03, B41,56, C01,17, DRB111,‐, DQB103,‐. These same results were observed for the donor. The patient underwent conditioning therapy with Busulfan and Fludarabine from days three to six before the transplantation. The investigation of possible germline alterations linked to the cancers was done using next‐generation sequencing (NGS). The Genome Analysis Toolkit (GATK) was used to do variant calling, referring to the GRCh37 genome build. Since there were no germline mutations connected to these tumors found by the NGS study, the patient does not have any distinguishable mutations that could account for his propensity to develop the cancers. Additionally, there were no incidental findings from the NGS data. Carrier screening analysis identified three pathogenic/likely pathogenic heterozygous mutations in the patient. These mutations were found in the following genes: ESAM, VWF, and NDUFV1. These mutations are listed in Table 1.

**TABLE 1:** Pathogenic and likely pathogenic mutations identified in the patient's carrier screening analysis.

Gene	Identified variant	Associated disease	Inheritance	Zygosity	ACMG/ClinVar classification
ESAM	Chr11 124628379 124628379 G ‐: NM_138961.3:(2/7): c.115delC:p.Arg39fs	Neurodevelopmental disorder with intracranial hemorrhage, seizures, and spasticity 620371	AR	Het	Pathogenic/likely pathogenic
VWF	Chr12 6128787 6128787 G T: NM_000552.4:(28/52):c.3797C>A:p.Pro1266Gln rs61749370	Von Willebrand disease, type 1 193400 Von Willebrand disease, type 3 277480 Von Willebrand disease, type 2A, 2B, 2M, and 2N 613554	AD AR AD, AR	Het	Likely pathogenic/conflicting interpretations of pathogenicity
NDUFV1	Chr11 67375913 67375913 G A: NM_007103.4:(2/10):c.119G>A:p.Arg40Gln rs141502688	Mitochondrial complex I deficiency, nuclear type 4 618225	AR	Het	Pathogenic/conflicting interpretations of pathogenicity

## 4 Discussion

### 4.1 Significant Findings and Challenges

This case report presents a unique and unprecedented clinical course in a 21‐year‐old nonhereditary retinoblastoma (RB) survivor who developed three sequential hematologic malignancies—acute myeloid leukemia (AML), lymphoma, and acute lymphoblastic leukemia (ALL)—over 20 years. The most significant finding is the occurrence of multiple, distinct hematologic secondary malignancies in a patient with no germline RB1 mutation or other known hereditary cancer predisposition. Nonhereditary RB survivors can demonstrate subsequent hematologic malignancies. A major challenge in this case was the absence of identifiable germline mutations, which complicated the assessment of cancer predisposition and risk stratification. Comprehensive next‐generation sequencing (NGS) and Sanger sequencing did not reveal any pathogenic variants in RB1 or other cancer predisposition genes, suggesting that the sequential cancers were likely driven by treatment‐related mutagenesis rather than inherited genetic factors. This underscores the importance of considering long‐term treatment effects, even in patients without a hereditary background.

### 4.2 Comparison With Previous Literature

In this article, we report three subsequent malignancies in a 21‐year‐old Iranian male who survived retinoblastoma. According to estimates, RB, the most common primary ocular malignancy in children and an aggressive disease, affects 1 in 16 000–20 000 live births worldwide. Both copies of the RB1 gene must be inactivated to lead to the development of either heritable or sporadic forms of retinoblastoma. In the hereditary form, individuals possess one defective germline allele and tend to have bilateral and multifocal tumors. However, in the sporadic form, both copies of RB1 are lost somatically [1, 2]. When diagnosed and treated early, the survival rate for RB patients can surpass 95%. But the danger of second and third malignant neoplasms later in life is a concern when ionizing radiation and chemotherapy are used as treatment modalities. The survival rate of RB patients has increased due to breakthroughs in diagnostic and treatment approaches; nonetheless, they are at a higher risk of acquiring and dying from second primary malignancies (SPCs) [5, 6, 7, 8, 12, 13]. Germline mutation carriers have a higher risk of SPCs, including pineoblastoma, osteosarcoma, soft tissue sarcoma, and melanoma, whereas carcinomas and hematological malignancies are less common [7, 22, 23]. The combination of genotyping susceptibility and treatment approaches influences the occurrence and type of SPCs in RB survivors [24]. The medical history of the present case revealed 3 sequential primary tumors during the 20 years of RB chemotherapy and radiotherapy. However, no mutations in the RB1 gene or other cancer predisposition genes were identified through whole blood whole‐exome sequencing analysis. Investigations demonstrated that radiotherapy and chemotherapy increase the risk of SPCs among RB survivors, and the risk is higher when both are used together [25]. Our patient was diagnosed with RB during infancy and developed AML as a secondary primary tumor 6 years after receiving chemotherapy and radiation therapy, and lymphoma and ALL were the next primary tumors 13 years later. The risk of SPCs is generally higher with the administration of external beam radiation (as in this case) compared to internal radiation techniques like plaque brachytherapy or intensity modulated radiation therapy. The use of specific chemotherapeutic agents like etoposide and alkylating agents, particularly in this case, would have resulted in a further increased risk of SPC [26]. Hematologic malignancies are very rare for third or subsequent primary tumors, and RB individuals are at a higher risk of developing fourth and fifth main tumors, with a median delay to third primary tumors of 5.8 years after SPCs [27]. Finally, our patient underwent bone marrow transplantation from his full‐match sibling. The survival rate of RB patients has increased over the years, despite the consistent incidence of SPTs, which may be linked to improved treatment methods for SPTs [28]. At 50 years post‐RB diagnosis, the total mortality from SPTs is 25% for patients with genetic RB, whereas it is 1% for those with non‐genetic RB [8]. S‐AML is thought to have a worse prognosis than de novo AML because it frequently relapses, has lower remission rates, and is generally more resistant to chemotherapy. So, the preferred therapy is allogeneic hematopoietic stem cell transplantation, but the overall prognosis remains poor [29]. Research shows that retinoblastoma survivors, especially those who underwent ionizing radiation therapy, have a higher risk of developing second and third malignancies compared to other cancer survivors [21]. Hereditary retinoblastoma, a rare form, accounts for 45% of cases and increases the risk of SMNs, whereas nonhereditary retinoblastoma, a somatic mutation, has a lower risk [30]. Retinoblastoma treatments have improved survival rates, but the associated risks of other tumors due to ionizing radiation remain a significant concern. The underlying mechanisms linking ionizing radiation exposure to the development of subsequent malignancy are still being elucidated. Ionizing radiation can cause DNA damage, which may lead to mutations in critical genes involved in tumor suppression and cell cycle regulation [31, 32]. Irradiation has been significantly associated with an increased risk of SPCs, and sarcomas are more frequently found in the irradiated periorbital field, while systemic diseases like leukemia are less likely [8, 12, 14, 33]. Hereditary survivors have a cumulative incidence of about 28% for any SMN after 40 years. The chance of acquiring SMNs is significantly reduced for survivors of nonhereditary retinoblastoma, with an SIR of 1.86, which is not statistically significant when compared to the general population. In contrast to approximately 5% of nonhereditary survivors, roughly 33% of hereditary RB survivors get at least one new malignancy 50 years after diagnosis. For nonhereditary survivors, SMNs increase most significantly after 40 years [20]. So the risk of second tumors is markedly higher in hereditary retinoblastoma survivors compared to their nonhereditary counterparts, driven by genetic predispositions and treatment‐related factors [8]. Nonhereditary RB survivors typically show few instances of SMNs before the age of 20 [20]. The most rapid increase in risk occurs after 40 years post‐diagnosis, with a cumulative incidence of around 5% at age 50. The overall risk for SMNs remains significantly lower in nonhereditary survivors compared to their hereditary counterparts throughout their lifespan [24]. Although chemotherapy is generally considered to have a lower associated risk of SMNs compared to radiotherapy, certain chemotherapeutic agents have been linked to specific risks. Etoposide, for instance, has been linked in certain cases to secondary acute myelogenous leukemia (AML). Certain chemotherapeutic drugs, particularly alkylating agents, have been linked to an increased risk of AML. AML may have developed as a result of chemotherapy's mutagenic effects, which can damage DNA and result in secondary cancers. Additionally, the genetic predisposition due to mutations in the RB1 gene may further contribute to this risk. Similar to AML, ALL can also occur as a secondary malignancy in RB survivors, although it is less commonly reported than AML [15, 26, 34]. Patients treated with chemotherapy might be at an increased risk of leukemia and lymphoma, and its leukemogenic effect is reported in multiple cohorts [16, 35]. Leukemia and lymphoma rarely happen as SPCs in RB patients, of which AML has the highest incidence, whereas ALL and lymphoma happen less frequently [14, 36, 37]. Nonetheless, the small number of patients and lack of long follow‐up time interfere with certain SPCs risks regarding chemotherapeutic agents in prolonged RB survivors. Furthermore, former studies mainly focused on solid SPCs due to the small occurrence rate of hematologic SPCs. Overall, while the prognosis for retinoblastoma has improved dramatically, the associated risks of subsequent SPCs remain a critical concern [7]. Continuous monitoring and tailored follow‐up care are essential for managing these long‐term risks in survivors. As a result, this case presentation emphasizes the importance of long‐term follow‐up and regular investigation of signs and symptoms of all heritable and nonheritable RB patients for early detection and urgent appropriate intervention to enhance life expectancy.

### 4.3 New Insights and Implications

This case expands the current understanding of SPC risk in RB survivors by demonstrating that nonhereditary patients are not immune to the late mutagenic effects of chemotherapy and radiotherapy. It suggests that the spectrum of SPCs in RB survivors may be broader than previously appreciated, and that long‐term, individualized surveillance is warranted regardless of hereditary status. Furthermore, our findings support the need for ongoing research into the mechanisms of treatment‐induced mutagenesis and the development of safer therapeutic protocols for RB. Personalized follow‐up strategies, including periodic hematologic evaluation and genetic counseling, are essential for early detection and management of SPCs in all RB survivors.

## Author Contributions

**Elham Karimi**, **Shalaleh Abbasnezhad**, **Maedeh Arabpour**, and **Sepideh Abdollahi:** conceptualization (equal), methodology (equal), resources (equal), and writing – original draft (equal). **Mohammad Biglari:** formal analysis (lead), software (lead), and writing – review and editing (lead). **Marjan Yaghmaie:** project administration (lead) and supervision (lead).

## Funding

This study was supported by the Research Institute for Oncology, Hematology and Cell Therapy (RIOHCT) affiliated with Tehran University of Medical Sciences (TUMS), grant (No. 1403‐1‐460‐70745).

## Consent

Informed consent for publication is obtained from the patient.

## Conflicts of Interest

The authors declare no conflicts of interest.

## Data Availability

The data that support the findings of this study are available from the corresponding author upon reasonable request.
